# Negative affordance effect: automatic response inhibition triggered by handle orientation of non-target object

**DOI:** 10.1007/s00426-021-01600-8

**Published:** 2021-09-25

**Authors:** L. Vainio, K. Tiippana, T. Peromaa, C. Kuuramo, I. Kurki

**Affiliations:** 1 Phonetics and Speech Synthesis Research Group, Department of Digital Humanities, University of Helsinki, Unioninkatu 40, Helsinki, Finland; 2 Perception, Action and Cognition Research Group, Department of Psychology and Logopedics, Faculty of Medicine, University of Helsinki, Haartmaninkatu 3, Helsinki, Finland

## Abstract

Habituated response tendency associated with affordance of an object is automatically inhibited if this affordance cue is extracted from a non-target object. This study presents two go/no-go experiments investigating whether this response control operates in response selection processes and whether it is linked to conflict-monitoring mechanisms. In the first experiment, the participants performed responses with one hand, and in the second experiment, with two hands. In addition, both experiments consisted of two blocks with varying frequency of go conditions (25%-go vs. 75%-go). The non-target-related response inhibition effect was only observed in Experiment 2 when the task required selecting between two hands. Additionally, the results did not reveal patterns typically related to conflict monitoring when go-frequency is manipulated and when a stimulus–response compatibility effect is examined relative to congruency condition of the previous trial. The study shows that the non-target-related response inhibition assists hand selection and is relatively resistant to conflict-monitoring processes.

**Supplementary Information:**

The online version contains supplementary material available at 10.1007/s00426-021-01600-8.

## Introduction

Grasping a handle is one of the most frequent habituated actions in everyday behavior. Typically, a handle is grasped with the hand that is compatible with the orientation and function of the handle (e.g., grasping a door handle with the hand towards which the handle is pointing). Anatomically, handle-directed grasping behavior is largely based on the fronto-parietal network in which certain parietal components extract affordances of perceived objects (i.e., properties of an object that implicitly define how the object can be used and grasped) and link this affordance information to corresponding motor representations (Fagg & Arbib, 1998; Thill et al., 2013). This network prepares handle-related grasp actions relatively automatically so that even the handle of a passively perceived object can trigger grasp motor activation of the hand that is compatible with the handle orientation (Bolton et al., 2019; Buccino et al., 2009; Cardellicchio et al., 2011).

The connections between handle perception and manual motor processes have been commonly investigated using a choice reaction time task in which participants are required to respond (e.g., press a key) with the left or right hand according to the category of the presented object (e.g., a kitchen or a toolbox item). In this task, responses are typically executed relatively rapidly and accurately when the target object has a handle whose orientation is compatible with the responding hand (the positive handle affordance effect) (McBride et al., 2012; Tucker & Ellis, 1998; Vainio et al., 2007). Electrophysiological research suggests that this effect is based on automatic and hand-specific motor activation compatible with the handle orientation of the target object (Goslin et al., 2012).

An opposite pattern of results is observed in relation to a handle of a perceived non-target object. This occurs in a task in which participants are required to respond with the left or right hand according to the pointing direction of the target arrow. When a non-target object is presented prior to or simultaneously with the target, responses are slowed down if the handle orientation of the non-target object is compatible with the responding hand (the negative handle affordance (NHA) effect) (Vainio, 2021; Vainio et al., 2011, 2014). Research suggests that the NHA effect reflects automatic inhibition of the response that is compatible with the handle orientation of a perceived non-target object in the absence of any facilitation of an incompatible response (Vainio, 2021; Vainio et al., 2014). This perspective is in line with the views that inhibition of response tendencies that are inappropriate to the ongoing behavior is crucial to optimally execute goal-directed behavior (Caligiore et al., 2013; Cardellicchio et al., 2018; Duque et al., 2017; Houghton & Tipper, 1994; Vainio & Ellis, 2020).

The processes that block habituated responses associated with affordances of non-target objects are far from understood. Investigation of the mechanisms underlying the NHA effect can improve understanding of these processes that control habitual behavior. Previously, the NHA effect has been speculated to be based on response conflict-monitoring processes (Vainio & Ellis, 2020). In addition, it has been suggested that the NHA effect is based on response control mechanisms that operate to solve potential conflicts of *response selection* caused by perceived affordance of a non-target object (Vainio, 2021). Although conflict-monitoring processes (Botvinick et al., 2001) have been established in particular to control stimulus-driven response selection (Correa et al., 2009), the conflict-monitoring processes can be also dissociated from response selection (Lau et al., 2006). More precisely, although conflict monitoring has been shown to control response facilitation automatically triggered by the prime stimulus (Ridderinkhof, 2002), whether conflict-monitoring processes also modulate response inhibition automatically triggered by the prime, i.e., the mechanism underlying the NHA effect (Vainio, 2021), remains unknown. Therefore, this study has two primary research questions that can be, but are not necessarily, inseparable: (1) whether the NHA effect operates within the hand-selection processes and (2) whether it is indeed based on conflict-monitoring processes.

## Is the NHA based on hand-selection processes?

Response selection is assumed to be based on the fronto-parietal network in which the parietal cortex represents possible responses (e.g., two hands), and which processes learned stimulus-response associations that can be activated by environmental cues (such as affordances) in a bottom–up fashion (Bunge et al., 2002). In contrast, specific prefrontal cortex (PFC) regions (e.g., lateral prefrontal and rostral anterior cingulate cortices) are important for selecting among competing response alternatives (Bunge et al., 2002; Leung et al., 2000). Response selection can be considered one of the earliest steps of action planning. Programming other elements of action planning, such as kinematic parametrization and planning the muscular force, cannot be initiated before the response is selected (Bernier et al., 2012). One of the most frequent daily decisions people make is selecting which hand to use for an action. These hand-selection processes are influenced by various aspects of the target object. For example, people preferably use the hand that is closer to the target when they reach for it (Oliveira et al., 2010), and handle orientation of an object can automatically bias selection of the hand for grasping an object, as stated above. Hand selection processes are prevented in situations in which only one hand is available, for example, when an individual has to open the door while one hand holds a bag or when grasping a jug, while the other hand holds a glass. In these situations, visually driven action planning has to mostly be carried out without the involvement of hand selection.

Previous research shows that behavioral effects that have been used to explore automatically operating connections between a stimulus and response (i.e., S–R compatibility effects) are typically based on response selection mechanisms. For instance, the Simon effect (Simon, 1969), which shows facilitated responding with the hand that is compatible with the left–right location of the target stimulus, is either eliminated or largely diminished when the task requires responding with one hand instead of selecting between two hands (Hasbroucq et al., 1988; Hommel, 1996). In fact, the influence of selective response preparation on the effects of response conflict has mostly been investigated using the Simon task. The general view is that the Simon effect is attributable to interference at the response selection so that task-irrelevant stimulus location automatically activates a spatially corresponding response, which competes for response selection when the task-relevant stimulus feature calls for an opposite response (Kornblum et al., 1990). Potential for this stimulus-triggered response conflict disappears when the response is already selected prior to onset of the stimulus.

The NHA effect might be similarly based on processes that select the effector for the response. Based on the accounts of action selection discussed above, the NHA can be observed when response readiness is approximately equal in both hands prior to stimulus onset and selection for action competes between these two response alternatives. When an individual is prepared to select the responding hand according to the target, as the task requires, a non-target that appears simultaneously with or in a close temporal proximity to the target is treated as a distractor by the fronto-parietal response selection processes. Consequently, representations associated with the non-target object—including response-related representations—are automatically inhibited, and hence, selecting a hand for the response that would be correct for the handle orientation but potentially incorrect for a current task is blocked. This in turn delays responses performed with the inhibited hand. This response selection account predicts that the NHA should be diminished or entirely removed when responses are performed with one hand because the handle affordance of the non-target cannot provide any biasing input to the response selection as these selection processes are entirely excluded from the task.

However, in some specific cases, S–R conflict effect has been observed even when response selection is not involved. For instance, in the study of Brass et al. (2001), participants performed pre-instructed finger movement (e.g., lifting) in response to the onset of a visually presented compatible (lifting) or incompatible (tapping) finger movement. There was a clear S–R compatibility effect between the movement of the stimulus and the movement of pre-instructed finger movement. Brass et al. proposed that when there is high ”ideomotor” compatibility between stimulus and response (i.e., stimulus or idea is capable of directly and involuntarily activating corresponding motor representation without requirement for S–R translation processes), compatibility effects can be observed in a task with minimal response selection requirements. Moreover, it is noteworthy that S–R compatibility effects based on purely spatial aspects of the stimuli (as in the Simon task) and imitative compatibility (as in the task of Brass et al.) appear to be attributable to different mechanisms (Weller et al., 2019).

Handle affordance of a viewed object can directly and involuntarily activate the motor representation of a hand that is compatible with the handle orientation even when no response is required (Bolton et al., 2019; Buccino et al., 2009; Cardellicchio et al., 2011). Hence, in the NHA effect, reflecting the ideomotor compatibility hypothesis, it is possible that the handle affordance of the non-target object triggers immediate motor inhibition of the hand representation that is compatible with the handle orientation. As a consequence, when the target arrow requires the response with this same hand, responding is slower than in the condition in which the handle orientation is incompatible with the responding hand. In line with this possibility, Pavese and Buxbaum (2002) reported that handle affordance of a distractor object can interfere with a single hand reaching for and grasping the handle of the target object. Hence, according to the ideomotor account, the NHA effect is not based on hand-selection processes, and consequently, the effect is observed regardless of whether the task is performed with one or two hands.

Finally, in some special circumstances, spatial S–R compatibility effects such as the Simon effect can be observed even when responding to the target does not require response selection at the time of the onset of the prime stimulus. The Simon effect can be observed in a single-response task in which participants are prepared to respond with two hands, but the responding hand is pre-cued prior to the onset of the spatial prime object (Hommel, 1996). The Simon effect can also be observed in a single-hand go/no-go block if it is carried out after the experimental block in which responding in the go/no-go task requires selecting between two hands (Ansorge & Wühr, 2004). However, in that study, the effect was entirely absent when the single-hand block was carried out before the two-hand block. This evidence suggests that response selection plays an important role in the effect, but also that pressure for response selection does not have to be an online property of the task; processes that are related to response selection in the Simon task can provide a carry-over effect from the two-hand task to the single-hand task. Due to this, the current study used a design in which half of the participants carried out only the two-hand S–R task, while half of the participants carried out only the single-hand task S–R task.

## Is the NHA based on conflict-monitoring processes?

The conflict-monitoring system, which is presumably located in the anterior cingulate cortex, provides an adaptive response control mechanism to monitor occurrences of stimulus-driven response conflict (Botvinick et al., 2001; Carter et al., 1998). A response conflict occurs when two or more conflicting response tendencies are simultaneously activated, and response selection must overcome interference from the prepotent but incorrect response tendency. Once such conflict is detected, the conflict-monitoring system conveys an alerting signal to the central control mechanisms of the PFC, informing these mechanisms of the need to become more vigilant to prevent future conflict.

Empirical evidence of these conflict-monitoring mechanisms is most frequently provided by congruency sequence effects (CSEs). The CSEs have been investigated in various S–R conflict paradigms, such as Simon task (Stürmer et al., 2002) and Flanker tasks (Gratton et al., 1992). These CSEs are often assumed to present conflict adaptation in terms of decreased S–R compatibility effects (i.e., greater recruitment of cognitive control) in post-conflict trials (Kunde & Wühr, 2006; Stürmer et al., 2002).

Response conflict can also be empirically manipulated by including low-frequency and high-frequency S–R conditions. Response conflict is enhanced in low-frequency S–R conditions to overcome the bias towards executing the prepotent response tendency related to high-frequency S–R conditions (Braver et al., 2001). For instance, regarding a go/no-go paradigm (see Verbruggen & Logan, 2008), if the block contains substantially more go-trials (e.g., 80%) than no-go trials, conflict adaptation should be enhanced relative to post-no-go trials. Correspondingly, if the block contains substantially more no-go trials, conflict adaptation should be enhanced relative to post-go trials (Donkers & van Boxtel, 2004; Nieuwenhuis et al., 2003). This predicts a reduced S–R compatibility effect in the trials after the low-frequency trials.

Similarly to most of the S–R compatibility effects, the NHA effect could be linked to these conflict-monitoring processes. Regarding the NHA effect, the conflict-monitoring mechanisms could detect response inhibition triggered by the handle orientation of a non-target and temporarily enhance response control processes for the subsequent trial, reducing the NHA effect in that trial. Importantly for this hypothesis, the handle affordance effect (i.e., response facilitation triggered by handle orientation of a target object) has been also shown to diminish in post-conflict trials (McBride et al., 2012). However, it has not been investigated whether automatic inhibition triggered by affordance of a non-target is similarly linked to these conflict-monitoring processes. It should be emphasized that conflict monitoring might be largely functioning to decrease stimulus-driven facilitation of a stimulus-compatible response of the subsequent trial (Ridderinkhof, 2002). However, the NHA effect has been shown to be based on response conflict related to inhibition of response compatible with the prime (Vainio, 2021), instead of increased activation of response compatible with the prime, as in typical S–R compatibility effects. Hence, it is equally possible that the NHA is based on different response control processes than typical S–R compatibility effects, and consequently, is not linked to conflict-monitoring processes.

## This study

Regarding the first research question of whether the NHA effect is based on response selection, the study includes two experiments: in the first experiment, responses are performed with one hand only, and in the second experiment responses are performed with two hands. Thus, the task of the first experiment does not recruit hand-selection processes, whereas the task of the second experiment does recruit hand-selection processes. If the NHA effect is observed in both experiments, it would suggest that the NHA effect is not based on hand-selection mechanisms, but if the effect is only observed in the second experiment, it would suggest that the effect is largely based on hand-selection mechanisms.

Both experiments employed the go/no-go paradigm in which the target is sometimes an arrow requiring a response and sometimes a line requiring response to be withheld. This set-up ensures that the participants must discriminate the target stimulus also when only one hand is used to respond. The experiments would not be comparable if both included only go (i.e., arrow) targets because in that case, responses of the first experiment would be based on target detection, whereas responses of the second experiment would be based on target discrimination.

With respect to the second primary research question, the study investigates the conflict-monitoring hypothesis by exploring the CSE of the NHA effect and by manipulating frequencies of go-trials within the experiments. Hence, both experiments consist of one block with a low frequency of go-trials (25%-go) and one block with a high frequency of go-trials (75%-go). It was assumed that if the NHA effect is linked to conflict-monitoring processes, it should be reduced in post-conflict trials (i.e., in trials after S–R compatible trials) relative to post-no-conflict trials (i.e., in trials after S–R incompatible trials) (note that in the case of the NHA effect, S–R compatibility refers to compatibility between the responding hand and the handle orientation of a non-target). It is also expected that the NHA effect should be reduced in the 75%-go block because that block contains more frequent conflict trials (i.e., S–R compatible trials) than the 25%-go block. Additionally, in the 75%-go block, the NHA effect should be smaller in post-no-go trials than in post-go trials. By contrast, in the 25%-go block, the NHA effect should be smaller in post-go trials than in post-no-go trials. This is because response conflict is enhanced relative to low-frequency response to overcome the bias towards executing the prepotent response tendency.

## Experiments 1 and 2

### Method

#### Participants

Sixty-one participants who had normal or corrected-to-normal vision participated in the study. Twenty-nine participated in Experiment 1 (5 males; 19–51 years of age; mean age = 23.5 years; all right-handed) and thirty-two participated in Experiment 2 (5 males; 17–49 years of age; mean age = 23.1 years; 2 left-handed). Our sample size calculation (estimated total sample size = 6), carried out using G*power software, was based on the results of that study (Vainio, 2021—Experiment 1), which showed a significant compatibility effect using 15 participants (*p* < 0.001; Cohen’s *d*_*z*_ = 1.4). The study was executed as a part of the course work in experimental psychology. Hence, participants were informed that the purpose of the experiment was to explore the NHA effect, and they understood that the NHA effect reflects relatively slow responses performed with the hand that was compatible with the handle orientation of the non-target. All gave their informed consent prior to their inclusion in the study. The study protocol was approved by the Ethics Review Board in Humanities and Social and Behavioural Sciences at the University of Helsinki.

### Apparatus, stimuli, and procedure

The study was carried out using a tablet computer^1^ (Apple iPad Air 3; operating system: iPadOS 14.2.) with 21.3 × 16.0 cm display (maximum luminance 500 cd/m^2^; screen refresh rate: 60 Hz; screen resolution: 2224 × 1668 pixels). The StimuliApp software (version 1.6) was used to run the study (Marin-Campos et al., 2020). The tablet was located horizontally on a table using a tablet cover as a stand. The participants were instructed to sit in a quiet room. The viewing distance was 50 cm from the tablet, measured using a measuring tape.

The stimuli consisted of the light gray fixation point (1.3°) and the target, which was the black line (no-go stimulus) or arrow (go stimulus) superimposed over the fixation point. The arrow was pointing to the left or right. The prime stimulus consisted of the image of a jug (subtended by a visual angle of 13° vertically and 9.5° horizontally), which was also used as a stimulus in the previous study (Vainio, 2021). The handle of the jug was pointing towards the left or right hand. (The stimuli can be delivered upon request.) All stimuli were displayed on a white background at the center of the screen. The jug was centered similarly to the study reported by Vainio (2021) such that the center point of the main body of the jug was at the center of the screen.

In Experiment 1, the participants responded with their right hand, while in Experiment 2, they performed responses using both hands. In Experiment 2, the hands were resting on a table so that the fingers were touching the back of the tablet, and the thumb of both hands was ready to respond by touching the screen. Therefore, the participants were instructed to keep their thumbs at a close distance to the screen. In Experiment 1, the method was similar to that of Experiment 2, with the exception that responses were given by the thumb of the right hand, while the left hand rested passively on the table next to the tablet.

**Fig. 1:**
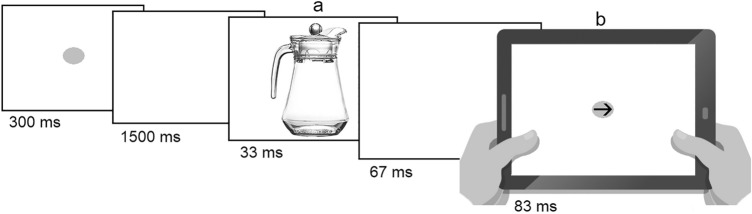
Schematic depiction of the structure of Experiments 1 and 2. The frame “a” presents the non-target prime, which was a jug whose handle was oriented to the left or right, and the frame “b” presents the target, which was a line (no-go stimulus) or the arrow (go stimulus). In Experiment 1, the arrow was oriented to the right, whereas in Experiment 2 the arrow was oriented to the left or right

Each trial started with the presentation of a fixation point. The point was displayed for 300 ms. Then the point was replaced by an empty white screen displayed for 1500 ms. Next the prime stimulus appeared on the screen for 33 ms. Then the prime was displaced by an empty white screen for 67 ms. Finally, the target appeared on the screen for 83 ms. The targets as well as the left- and right-oriented prime objects were presented in random order with equal probability. A blank white screen was displayed until the participant responded or the trial timed out 700 ms after target offset. The timing was adapted from Vainio (2021), with slight modifications to accommodate for the lower refresh rate (60 Hz) of the display compared to the previous study. Figure 1 shows the trial structure.

In Experiment 2, the participants were instructed to respond with their right thumb if the arrow was pointing to the right and with their left thumb if it was pointing to the left. One-third of the left and right sides of the screen were programmed to collect responses. The participants were instructed to respond as quickly as possible, while maintaining accuracy, when the arrow was presented as a target (go-trials). They were instructed to withhold responding when the line was presented as a target (no-go trials). The structure of Experiment 1 was similar to that of Experiment 2, with the exception that the left-pointing arrow was not included. In this way, the go and no-go conditions of Experiment 1 and 2 were as identical to each other as possible, with the only difference that in Experiment 2, the go stimuli required selecting between the left and right hand. In Experiment 1, the participants were instructed to touch the right side of the screen with their right thumb as a response to the right-pointing target arrow and withhold their response if the line was presented as a target.

Both experiments started with an 8-trial practice, where 50% of the trials were go-trials. Both experiments consisted of two blocks that included different portions of go-trials. The order of the blocks was counterbalanced between the participants. In one block (75%-go), 75% of the trials were go-trials and 25% were no-go trials. In another block (25%-go), 25% of the trials were go-trials and 75% were no-go trials. Given that the 25%-go block included three times fewer go trials than the 75%-go block, the 25%-go block consisted of three time more trials than the 75%-go block to collect the same number of data points (i.e., go responses) in both blocks. The 25%-go block included two breaks, while the 75%-go block did not include any breaks. The participants were free to rest during the breaks for a minimum of 15 minutes to avoid any unnecessary fatigue.

The 75%-go block of Experiment 1 consisted of 96 go-trials in which each stimulus was displayed 48 times in each condition (left handle/right arrow, right handle/right arrow) and 32 no-go trials in which each stimulus was displayed 16 times in each condition (left handle/line, right handle/line). The 25%-go block of Experiment 1 consisted of 96 go-trials in which each stimulus was displayed 48 times in each condition (left handle/right arrow, right handle/right arrow) and 288 no-go trials in which each stimulus was displayed 144 times in each condition (left handle/line, right handle/line).

In total, the 75%-go block of Experiment 2 consisted of 96 go-trials in which each stimulus was displayed 24 times in each condition (left handle/left arrow, left handle/right arrow, right handle/left arrow, right handle/right arrow) and 32 no-go trials in which each stimulus was displayed 16 times in each condition (left handle/line, right handle/line). The 25%-go block of Experiment 2 consisted of 96 go-trials in which each stimulus was displayed 24 times in each condition (left handle/left arrow, left handle/right arrow, right handle/left arrow, right handle/right arrow) and 288 no-go trials in which each stimulus was displayed 144 times in each condition (left handle/line, right handle/line). The participants were not informed about the purpose of the frequency manipulation. In general, no instructions were given between the two blocks.

## Results

The no-go trials and errors [i.e., the participant responded with the wrong hand in Experiment 2 (2.3%) or did not produce any response in the go trial (Experiment 1: 0.2%; Experiment 2: 0.3%)] were excluded from the reaction time analysis. Reaction times were measured from the onset of the target arrow to the onset of the thumb press. Similarly to the original study (Vainio, 2021), reaction times slower than 800 ms (0.1%) and faster than 200 ms (0.2%) were excluded as anticipations. This lower cut-off level was selected because it typically takes a minimum of 200–300 ms to respond to a visually presented target (Welford, 1980).

### Reaction times

The reaction time data of Experiments 1 and 2 were analyzed in a single linear mixed model analysis. The analysis treated Frequency-block (25%-go, 75%-go), Experiment (one hand, two hands), and Compatibility between handle and arrow direction (compatible, incompatible) as fixed factors and Subject as a random intercept. Selection of error covariance structure was based on Schwarz’s Bayesian information criterion (BIC). All tests of pairwise comparisons were carried out using Bonferroni correction for multiple comparisons. The analysis was carried out using SPSS software package (version 27).

After estimating the best-fitting error covariance structure (BIC = 121855.49), the analysis of reaction times revealed significant main effects of Frequency-block [*F*(1,59) = 45.38, *p* < 0.001] and Compatibility [*F*(1,59) = 96.39, *p* < 0.001]. In addition, the analysis showed significant interactions between Frequency-block and Compatibility [*F*(1,11322) = 47.41, *p* < 0.001], Experiment and Frequency-block [*F*(1,59) = 9.16, *p* = 0.004], Experiment and Compatibility [*F*(1,59) = 66.55, *p* < 0.001], and Frequency-block, Experiment, and Compatibility [*F*(1,11322) = 21.11, *p* < 0.001]. The pairwise comparison test showed that when one hand was used (Experiment 1), the difference between compatible and incompatible responses was not significant in Frequency-blocks of 25%-go (*p* = 0.921) or 75%-go (*p* = 0.071). In contrast, when two hands were used (Experiment 2), the difference was significant (*p* < 0.001) in both frequency conditions.

To further explore the significant three-way interaction, the data of Experiments 1 and 2 were analyzed separately. In Experiment 1 (BIC = 59024.54), the main effect of Frequency-block was significant [*F*(1,28) = 57.05, *p* < 0.001]. Responses were faster in the 75%-go block (*M* = 354 ms) than in the 25%-go block (*M* = 391 ms). The main effect of Compatibility (*p* = 0.122) and the interaction between Frequency-block and Compatibility were not significant (*p* = 0.133). Moreover, when the Order of Frequency-block (1 = 25%-go first, 2 = 75%-go first) was added as a factor, the main effect of Order (*p* = 0.269), the three-way interaction of Order*Frequency-block*Compatibility (*p* = 0.297), and as well as the two-way interactions of Order*Frequency-block (*p* = 0.131) or Order*Compatibility (*p* = 0.160) were not significant. This suggests that the compatibility effect was absent regardless of which Frequency-block was the first block of the experiment.

**Fig. 2:**
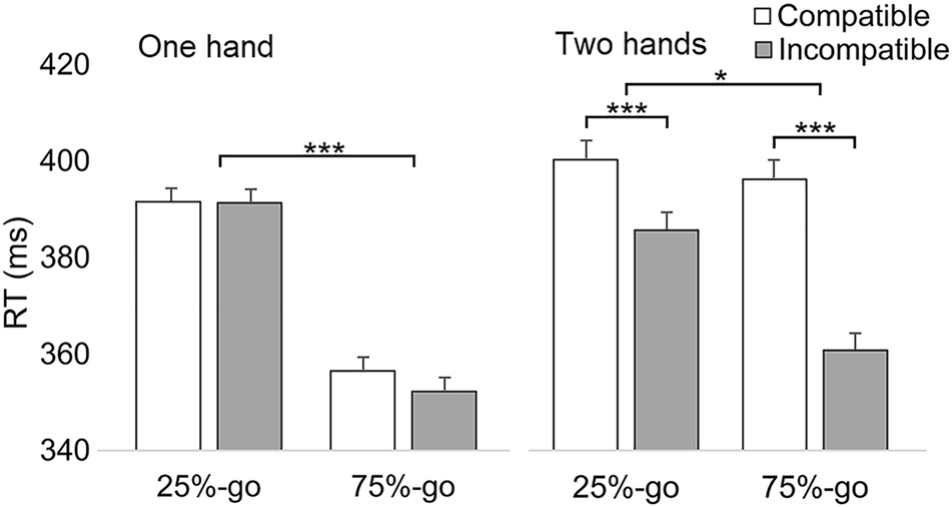
Reaction times (ms) for Experiments 1 and 2 (one hand vs. two hands) as a function of Compatibility (compatible vs. incompatible), and Frequency-block (25%-go vs. 75%-go). Error bars represent standard errors of paired differences for the comparison of Compatible and Incompatible, computed separately for 25%-go and 75%-go responses (Pfister & Janczyk, 2013). Asterisks indicate significant differences (***p < 0.001)

However, the interaction between Frequency-block and Compatibility was significant (*p* < 0.001) in Experiment 2 (BIC = 62775.48). Although the difference between Compatible and Incompatible responses was significant (*p* < 0.001) in both Frequency-blocks of Experiment 2, the NHA effect was larger in the 75%-go block (compatible: *M* = 395 ms, incompatible: 360 ms; mean difference (incompatible-compatible) = −35 ms, SE = 2.5, *d*_*z*_ = 0.61) than in the 25%-go block (compatible: *M* = 398 ms, incompatible: 384 ms; mean difference = −14 ms, SE = 2.5, *d*_*z*_ = 0.24). In addition, the main effect of Frequency-block was significant [*F*(1,31) = 6.1, *p* = 0.019]. Responses were faster in the 75%-go block (*M* = 377 ms) than in the 25%-go block (*M* = 392 ms). Moreover, when the Order of Frequency-block (1 = 25%-go first, 2 = 75%-go first) was added as a factor, the main effect of Order (*p* = 0.784), the three-way interaction of Order*Frequency-block*Compatibility (*p* = 0.362), and as well as the two-way interactions of Order*Frequency-block (*p* = 0.728) or Order*Compatibility (*p* = 0.785) were not significant. This suggests that the compatibility effect was present regardless of which Frequency-block was the first block of the experiment. Figure 2 shows that the NHA effect occurs only when responses are performed with two hands and that the effect is larger when the block contains a relatively large number of go-trials (75%-go) in Experiment 2.

### Conflict modulation

It was also examined whether the effect of compatibility on a given trial was modulated by the response condition of the preceding trial as in traditional “conflict” tasks, such as Simon (e.g., Wühr & Ansorge, 2005) and Eriksen flanker (e.g., Gratton et al., 1988). This analysis was carried out by following the instructions for conflict adaptation analysis expressed by Braem et al. (2019), i.e., the first trial of each block and all trials following an error (in addition to the actual error trials) were removed from the analysis. Only Experiment 2 was analyzed given that Experiment 1 did not reveal the NHA effect. The post-conflict trials (i.e., the trials after S–R compatible trials) and the post-no-conflict trials (i.e., the trials after S–R incompatible trials) were included in this analysis. In addition, given that low-frequency no-go trials have been associated with increased conflict (Braver et al., 2001), one might assume that the NHA effect would be particularly reduced in the post-no-go trials of the 75%-go Frequency-block. Hence, the post-no-go trials were also included in this analysis.

**Fig. 3:**
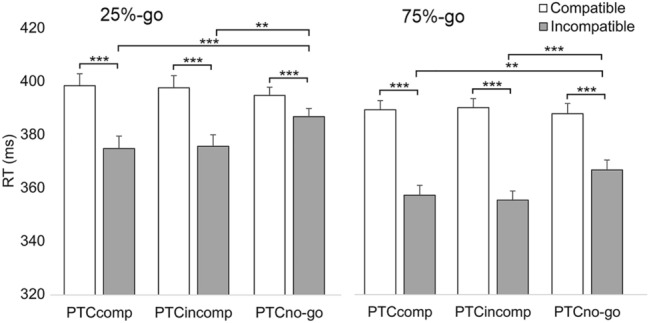
Reaction times (ms) for Experiment 2 showing the NHA effect (i.e., difference between compatible and incompatible responses) as a function of Frequency-block (25%-go, 75%-go) and PTC (previous trial condition; compatible, incompatible, no-go). Error bars represent standard errors of paired differences for the comparison of Compatible and Incompatible, computed separately for PTCcomp, PTCincomp and PTCno-go conditions. Asterisks indicate significant differences (***p < 0.001, **p < 0.01)

Figure 3 shows that the NHA effect is relatively small in both Frequency-blocks when the preceding trial is a no-go trial. Hence, the reaction time data of Experiment 2 were analyzed with fixed within factors of Frequency-block (25%-go, 75%-go), PTC (previous trial condition: compatible, incompatible, no-go), and CTC (current trial condition: compatible, incompatible). The analysis (BIC = 62624.85) revealed a significant two-way interaction between PTC and CTC [*F*(2,5887) = 11.19, *p* < 0.001], while the three-way interaction between Frequency-block, PTC, and CTC was not significant [*F*(2,5885) = 0.25, *p* = 0.779]. The pairwise comparison test showed that in both Frequency-blocks, incompatible responses were significantly slower when preceded by no-go trials than in compatible and incompatible trials (25%-go: PTCcomp *M* = 375 ms vs. PTCno-go *M* = 387 ms; mean difference = 12 ms, SE = 3.8, *p* = 0.007, *d*_*z*_ = 0.41; PTCincomp *M* = 376 ms vs. PTCno-go; mean difference = 11 ms, SE = 3.8, *p* = 0.010, *d*_*z*_ = 0.37; 75%-go: PTCcomp *M* = 358 ms vs. PTCno-go *M* = 368 ms; mean difference = 10 ms, SE = 3.3, *p* = 0.013, *d*_*z*_ = 0.38; PTCincomp *M* = 356 ms vs. PTCno-go; mean difference = 12 ms, SE = 3.4, *p* = 0.002, *d*_*z*_ = 0.46). However, the difference between Compatible and Incompatible responses (i.e., the NHA effect) did not differ between the post-conflict (PTCcomp) and the post-no-conflict (PTCincomp) conditions.

### Error rates

**Fig. 4:**
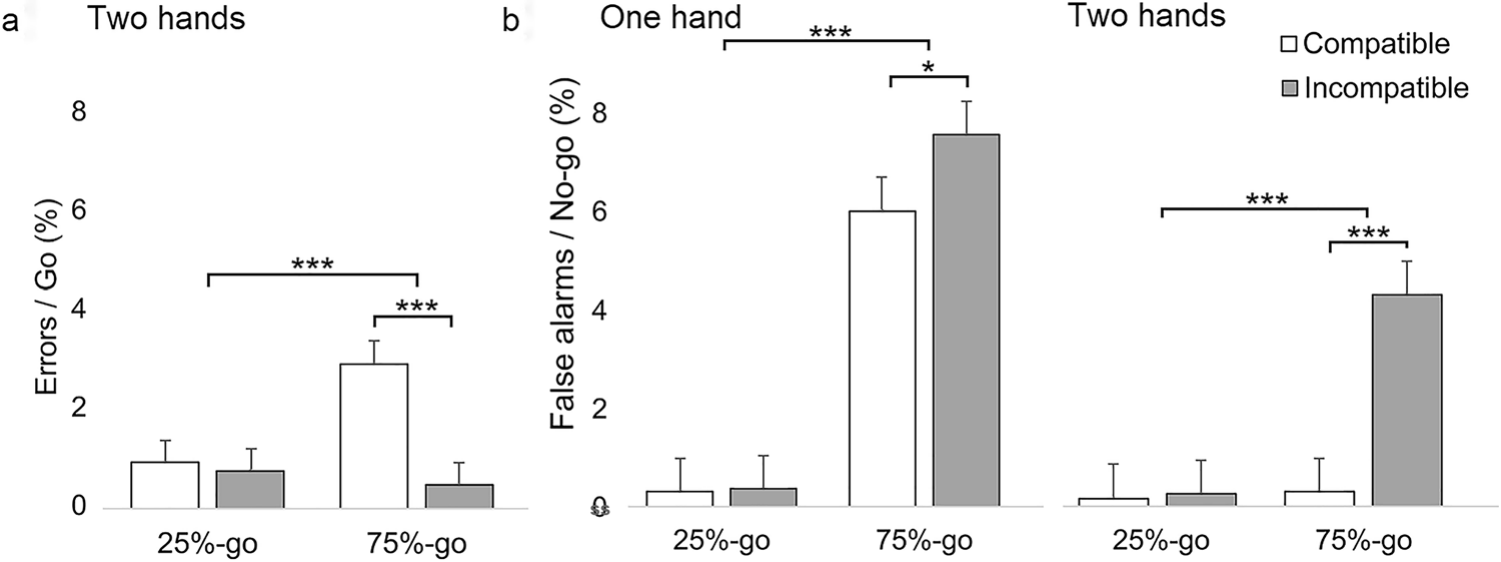
Percentage error rates. **a** Error rates for Experiment 2 as a function of Compatibility (compatible, incompatible) and Frequency-block (25%-go, 75%-go). High error rates signal that the participants show a tendency to respond with an incorrect hand in a given go-condition. **b** False-alarm rates as a function of Experiment (one hand, two hands), Compatibility (compatible, incompatible), and Frequency-block (25%-go, 75%-go). Error bars represent standard errors of paired differences for the comparison of Compatible and Incompatible, computed separately for 25%-go and 75%-go responses. Asterisks indicate significant differences (***p < 0.001, *p < 0.05)

The percentage error rates (i.e., participant responded with incorrect hand, shown in Fig. 4a) were analyzed for Experiment 2 using a single linear mixed model analysis that treated Frequency-block (25%-go, 75%-go), and Compatibility between handle and arrow direction (compatible, incompatible) as fixed factors and Subject as random intercept. Two participants did not make any such errors. The analysis (BIC = 448.66) revealed a significant main effects of Frequency-block [*F*(1,87) = 10.98, *p* < 0.001] and Compatibility [*F*(1,87) = 25.42, *p* < 0.001]. The participants made more errors in the 75%-go condition (*M* = 1.6%) than in the 25%-go condition (*M* = 0.8%) and in Compatible condition (*M* = 1.8%) than in Incompatible condition (*M* = 0.6%). In addition, the interaction between Frequency-block and Compatibility was significant [*F*(1,87) = 22.60, *p* < 0.001]. The participants made more errors in 75%-go condition when the handle orientation was compatible (*M* = 2.8%) rather than incompatible (*M* = 0.4%) with the required response (mean difference = 2.4%, *p* < 0.001, SE = 0.3, *d*_*z*_ = 0.93). However, in the 25%-go block, this effect was not significant (compatible: *M* = 0.8%, incompatible: *M* = 0.8%). That is, the participants preferred to respond with the hand that was incompatible with the handle even when the target required the compatible response, but only in the 75%-go block.

We also analyzed error rates when the no-go target required withholding the response (i.e., false-alarm responses) using a single linear mixed model analysis that treated Frequency-block (25%-go, 75%-go), Compatibility between handle and arrow direction (compatible, incompatible) and Experiment (one hand, two hands) as fixed factors and Subject as random intercept. Twelve participants did not make any such errors. In addition, the false-alarm data of one participant (participant number ten) were removed from this analysis because more than 10% of his/her no-go performances were false-alarm responses. The analysis (BIC = 995.44) revealed a significant main effects of Frequency-block [*F*(1,46) = 52.94, *p* < 0.001], Compatibility [*F*(1,46) = 15.83, *p* < 0.001] and Experiment [*F*(1,46) = 14.85, *p* < 0.001]. The participants made more false-alarm responses in 75%-go condition (*M* = 4.5%) than in 25%-go condition (*M* = 0.3%), in incompatible (*M* = 3.1%) rather than in compatible (*M* = 1.7%) condition, and in Experiment 1 (*M* = 3.6%) than in Experiment 2 (*M* = 1.3%). The two-way interaction of Frequency-block*Compatibility was also significant [*F*(1,46) = 16.32, *p* < 0.001]. The pairwise comparisons test showed that the participants produced more false-alarm responses in 75%-go condition when the handle orientation was incompatible rather than compatible with the produced false-alarm response in both experiments (one-hand compatible: *M* = 6.0%, incompatible: *M* = 7.6%; mean difference = 1.6%, SE = 0.7, *p* = 0.028, *d*_*z*_ = 0.21; two-hand compatible: *M* = 0.3%, incompatible: *M* = 4.3%; mean difference = 4%, SE = 0.7, *p* < 0.001, *d*_*z*_ = 0.64). In contrast, in 25%-go conditions of Experiments 1 and 2, false-alarm responses were not significantly different between compatible and incompatible conditions. These findings show that, in 75%-go conditions of Experiment 1 and 2, the participants were particularly prone to execute the response—even in the no-go conditions—with the hand that was incompatible rather than compatible with the handle even when the target called for withholding the response. This effect is presented in Fig. 4b. The analysis of the no-response data related to the go (misses) and no-go (correct rejections) trials did not reveal any effects.

## General discussion

The results of the present study revealed the NHA effect in the go/no-go task. Even though the task required responding to the target, responses were performed slower when the handle orientation of the non-target object was compatible rather than incompatible with the responding hand. This observation was supported by the results of error rates. The participants often preferred to respond with the hand that was incompatible with the handle even when the target called for the compatible response.

With respect to no-go trials, some participants showed more tendency to respond with the hand that was incompatible rather than compatible with the handle even when the target called for withholding the response. The observations related to errors of go and no-go trials present evidence for automatic inhibition of response that was compatible with the handle orientation of the non-target. Due to this inhibition, responding with the handle-compatible hand is temporally blocked, resulting in relatively frequent task-inappropriate responding in the go and no-go conditions with the opposite (i.e., not inhibited) hand.

Furthermore, the fact that the NHA effect can be observed even in the false-alarm errors related to the no-go trials underlines the response-related nature of the effect. This demonstrates that the effect cannot be based on purely perceptual processes. It would have been possible that the effect is based on, for example, inhibited perceptual processing of the target arrow, triggered by the prime handle, delaying discrimination of the arrow whose direction is compatible with the handle of the non-target. Instead, the effect is based on genuine handle-related response inhibition that can manifest itself also in false-alarm responses even when the target is a hand-neutral line requiring withholding the response.

Liu et al. (2016) have previously suggested that the NHA effect is observed because, in the task, participants intentionally avoid responding to the arrow with the hand that is compatible with the handle position of the prime. This conclusion was based on the finding that the NHA effect is reversed into a positive compatibility effect when the handle position is compatible with the direction of the target arrow in 80% of the trials, and participants are informed that they would improve their response speed and accuracy if they took this aspect into account when carrying out the task. However, the fact that the same NHA effect is observed even when the handle position does not provide any cue about the direction of the target arrow (i.e., when 50% of the trials are compatible and 50% are incompatible with the target arrow) (Liu et al., 2016; Vainio et al., 2011) suggests that the effect can be observed even when participants do not use the handle position as a strategic cue according to which they predict the direction of the upcoming arrow. This view is supported by several findings of Vainio (2021). Most importantly, Vainio (2021) showed that the NHA effect is observed even when only the first five compatible and incompatible responses are analyzed from each participant, and participants are not forewarned about the appearance of the prime.

The current study approached this automaticity question from a new angle by asking whether the NHA effect is observed even when the participants are fully aware that the best strategy to minimize any S–R conflict and maximize the speed and accuracy of responses is to ignore the non-target object. To understand the nature of the NHA effect, it is important to explore the influence of pre-knowledge on the effect because it has been shown that even automatically operating processes in S–R effects can be modulated by instructions (Meiran et al., 2017). The present study replicated the NHA effect even though all participants were aware of the purpose of the study. The same effect has been observed when none of the participants figured out that the handle orientation of the non-target could in any way influence responses (Vainio, 2021). In light of these results, it appears that the effect is resilient to pre-knowledge or lack of pre-knowledge about the influence of the non-target on responses. This supports previous observations (Vainio, 2021) that the effect is based on automatic response control processes that are difficult to strategically avoid.

One of the primary goals of the study was to investigate whether the NHA effect reflects response selection processes. In this respect, the outcome of the study is quite straightforward. The NHA effect was observed in reaction times, error rates, and false-alarm responses of Experiment 2 when the task required selection between two hands. In contrast, in Experiment 1, the effect was absent in reaction times and false-alarm responses when the participants knew in advance the hand that they should use for the response. It can be argued that the go/no-go task used in Experiment 1, in which a participant either executes the response with the right-hand (go trial) or withholds responding with that hand (no-go trial) also contains response selection demands. The participant must *select* whether to respond or withhold the response. Therefore, for the sake of being careful with terminology, we can state that the NHA effect is grounded at least in visually driven hand-selection processes that inhibit selecting the hand for the response that is appropriate to the affordance of a non-target object but potentially inappropriate to the current task.

Furthermore, one aspect to be considered when interpreting the current results is the influence of response readiness on the NHA effect. It has been shown that response readiness is increased when the probability of Go trials is high (80%), while a low probability of Go trials (20%) reduces response readiness (Low & Miller, 1999). Although the NHA effect was observed in 25%-go and in 75%-go conditions of Experiment 2, the effect was clearly larger in the 75%-go condition. This perspective is in line with the finding that response preparation boosts processing affordances of perceived objects (Symes et al., 2008). This together with the finding that the effect was eliminated in the single-hand task suggests that the NHA effect requires that the task includes a present pressure for hand/response selection, and that the optimal pre-condition for observing the effect is that response readiness is high.

Nevertheless, the fact that the participants produced significantly more false-alarm responses in incompatible condition even when the responses were performed with one hand suggests that although the NHA effect mostly operates within hand-selection processes, some minor ideomotor S–R mapping processes might also contribute to the effect. However, as compared to the NHA effect related to influence of non-target affordance on hand selection, these ideomotor S–R mapping processes might be too mild so that they could influence reaction times of keypresses performed with one hand. Perhaps the influence of this direct S–R mapping—related to non-target affordance—on responses of a single hand is particularly boosted when participants are required to actually reach and grasp the handle of the target object as observed by Pavese and Buxbaum (2002).

The second primary goal of the study was to explore whether the NHA effect is linked to conflict-monitoring processes. The results showed that the NHA effect is not modulated by the CSE (i.e., congruency sequence effect: reduced S–R compatibility effect in post-conflict trials). The NHA effect was not reduced (or increased) in the post-conflict trials relative to post-no-conflict trials, suggesting that it is not linked to conflict-monitoring processes. The only consistent post-trial effect that was observed in the study was that the NHA effect was reduced when the previous trial was a no-go trial. This effect was observed regardless of whether the no-go trial was the low- or high-frequency condition. Why then is the NHA reduced after no-go trials? The most likely explanation is that the mechanisms that enable withholding a response in the no-go trials of go/no-go tasks recruit response inhibition in addition to conflict-monitoring processes (Donkers & van Boxtel, 2004; Nieuwenhuis et al., 2003). It is possible that this no-go-related inhibition control also impacts the response control processes of the subsequent trial.

It is noteworthy that this assumed post-no-go inhibition primarily influenced the speed of handle-incompatible responses, i.e., the responses that are not inhibited by the handle orientation. These handle-incompatible responses were performed significantly slower after the no-go trials than after compatible or incompatible trials. It is plausible that response inhibition reflecting from the no-go condition provides increased response inhibition to the response control processes of the subsequent trial, slowing down in particular those responses that are not otherwise inhibited (i.e., the handle-incompatible responses). Perhaps this post-no-go trial inhibition does not have a similar influence on handle-compatible responses of the subsequent trial because response inhibition is already substantial in that S–R condition, resulting in a ceiling effect. That is, post-no-go response inhibition does not enhance or reduce the response inhibition triggered by handle orientation of a non-target object. Given that the CSE was also not observed to modulate the inhibition of handle-compatible responses, it seems that the mechanisms that inhibit responses compatible with affordance of a non-target are relatively resistant to the influences of the preceding trial. They operate in a here-and-now manner.

The current study shows that non-target-triggered response inhibition is based on response control processes that are somewhat distinct from those that control S–R conflict in typical S–R compatibility effects. This can be assumed because S–R compatibility effects are typically modulated by the CSE (Gratton et al., 1992; McBride et al., 2012; Stürmer et al., 2002), whereas the CSE was not observed in the present study. At least it seems that the NHA effect is largely immune to conflict adaptation as opposed to most of the S–R compatibility effects. The most salient way in which the NHA differs from most of the S–R compatibility effects is that the NHA effect reflects inhibition of response that is compatible with a non-target, in the absence of any facilitation of incompatible response (Vainio, 2021; Vainio et al., 2014). In contrast, most of the other S–R compatibility effects largely reflect facilitation of response that is compatible with stimuli, which in turn is inhibited if this facilitated response is inappropriate to the ongoing task. It is noteworthy that conflict adaptation is enhanced when the stimulus *facilitates* a task-inappropriate response (i.e., in S–R incompatible conditions), and consequently, the conflict adaptation mechanisms are likely, to a large extent, to function to reduce stimulus-driven facilitation of the stimulus-compatible response to the post-conflict trial (Ridderinkhof, 2002). If this is the case, it is not surprising that conflict adaptation does not influence the NHA effect in the post-conflict trial. As this conflict effect is based on response inhibition, there is no stimulus-triggered response facilitation that could be reduced by these response control processes of conflict adaptation.

In addition to linking response control processes related to the go/no-go tasks to conflict monitoring (e.g., Donkers & van Boxtel, 2004), even more commonly withholding responses in the no-go trials of the go/no-go tasks have been investigated to reveal response inhibition mechanisms (e.g., Van Boxtel et al., 2001). It has been previously proposed that the NHA effect might recruit the response inhibition mechanisms that overlap with the mechanisms enabling withholding response in the no-go trials of the go/no-go tasks (Vainio & Ellis, 2020). The results of the present study support this proposal to some extent. The fact that the post-no-go inhibition did not influence affordance-triggered inhibition of affordance-compatible responses suggests that these inhibitory phenomena can be based on overlapping mechanisms. However, if these phenomena were based on the same response inhibition processes, one might assume that we should have observed an inhibitory post-trial effect also after response inhibition associated with affordance-compatible responses. Although this was not observed, it is possible that response inhibition linked to no-go trials is so much more powerful (e.g., requiring voluntary withholding of response) than response inhibition linked to affordance-compatible responses that its post-trial influence can be only observed in the post-no-go trials. However, this question warrants further investigations.

Finally, when exploring influence of handle orientation on responses of left and right hand, one must verify that the effect is indeed based on functional handle affordance information instead of abstract lower-level visual features of the stimuli (see Anderson et al., 2002; Cho & Proctor, 2010; Proctor & Miles, 2014). This issue was verified in previous investigations by showing that the NHA effect was removed when a mug was replaced by a mug-like object whose horizontal spatial features (i.e., handle-related properties) were identical to that of the mug stimulus, but whose vertical spatial features were modified so that the participants did not recognize the object as a mug (Vainio et al., 2011). In line with that behavioral finding, electrophysiological investigation has shown that the pattern of hand motor activation observed with a mug-like object is opposite to that of a mug stimulus in an otherwise identical NHA task (Vainio et al., 2014). A more recent NHA study (Vainio, 2021) that used a jug as a non-target object—the same jug that was also used in the current study—verified this issue by showing that the NHA effect was removed when the jug was presented in an inverted position, even though the visual saliency bias associated with the handle orientation was identical in upright and inverted positions. These findings are in line with the observations of Riddoch et al. (1998), providing neuropsychological evidence to demonstrate that the handle information of the object loses its capacity to evoke responses when the functional meaning of the object is reduced by inverting it or when it is not recognized as a familiar graspable object. Hence, based on previous evidence, it can be concluded that the NHA effect observed in the current study is based on functional handle affordance information instead of abstract lower-level visual features of the stimuli.

## Limitations, alternative accounts and future directions

The current study did not employ an established protocol for measuring CSEs in the absence of learning and memory confounds (Schmidt & Weissman, 2014), so that the CSE-related findings of the study could be exclusively attributed to adaptive response control processes instead of feature integration processes. This can be taken as a limitation of the current study. Indeed, the exact processes that give rise to CSEs have remained controversial. In general, two different accounts have been offered to CSEs. The gating account explains them by enhanced cognitive control after the occurrence of a response conflict (e.g., Kunde & Wühr, 2006; Stürmer et al., 2002). In contrast, the feature integration account assumes that CSEs originate from unequal repetitions of response and/or stimulus features across different congruency sequences rather than being a consequence of cognitive control operations (Hommel et al., 2004; Mayr et al., 2003). Nevertheless, the fact the the CSE was absent in the current study regardless that this potential confound was not controlled for might be taken to strengthen rather than weaken our conclusion that the NHA effect is not modulated by sequency factors.

Moreover, as already discussed elsewhere (Vainio & Ellis, 2020), it is possible that instead of being based on response inhibition processes, the NHA effect might be also explained by the theory of event coding (TEC) (Hommel et al., 2001; Hommel, 2004). The TEC would assume that the NHA effect is based on automatic integration of the perceptual code (e.g., the left position of a handle) and the response code (e.g., left response) associated with the prime stimulus. According to this view, responding is hampered if the target stimulus is presented immediately after the offset of the handle prime and the target calls for recruiting the response code that is already engaged to sensorimotor processing of the prime object (i.e., when the handle position of the prime is compatible with the response required by the target arrow). Although it is not clear how the TEC would explain, for example, the findings that the upright jug primes result in the NHA effect while the effect is absent with the inverted jug primes (Vainio, 2021), this framework should be still explored as an explanation for the NHA effect that is alternative to the response inhibition explanation.

Regarding possible future directions, for the sake of further exploring whether the NHA effect is based on different or overlapping sensorimotor mechanisms to those of spatial S–R compatibility effect such as the Simon effect, future studies should investigate whether the NHA effect is absent in an experimental block that requires responding with one hand, as in Experiment 1, but that is carried out directly after the experimental block in which responses are performed with two hands. Indeed, in these circumstances, the Simon effect is observed even when responses are performed with a single hand, suggesting that working memory representations of the left and right responses constructed during the first block can carry over to the subsequent single-response block (Ansorge & Wühr, 2004). Moreover, the Simon effect can be observed in a social Simon task in which the same single-hand go/no-go task is shared between two participants in the manner that each participant operates one of the two responses (Dolk et al., 2014; Sebanz et al., 2003). It would be interesting to explore whether the NHA effect can be similarly observed in a corresponding single-hand version of the joint S–R compatibility task. Finally, it has been shown that the positive handle affordance effect (Tucker & Ellis, 1998) can be observed when left and right responses are performed with the middle and index finger of a single hand (Vainio et al., 2007). Hence, for the sake of investigating whether the NHA effect is based on the same response control processes as the positive handle affordance effect, it should be investigated whether the NHA effect can be similarly observed in a corresponding single-hand task.

In conclusion, the study showed that the mechanisms inhibiting the response associated with handle orientation of a non-target object operate in hand-selection processes and are mostly resistant to conflict adaptation processes. Handle orientation of a distractor object automatically inhibits selection of a hand that would be appropriate to the orientation but potentially inappropriate for the ongoing task. This response inhibition influences behavior only at the time of the conflict, without being registered by top–down conflict-monitoring processes. This finding is in line with the results suggesting that the NHA effect is based on habituated response control processes that have developed over a lifetime rather than sequential or other task-related factors associated with the experiment (Vainio, 2021). In general, the NHA effect together with the positive handle affordance effect (Tucker & Ellis, 1998, 2001) suggests that response control processes that program habitual responses related to object affordances consist of automatically operating facilitatory and inhibitory mechanisms. If affordance information is associated with a target object that calls for a response, it facilitates the response that is compatible with it (Tucker & Ellis, 1998, 2001). As a compensation for this, if affordance information is associated with a non-target that requires withholding response, it inhibits the response that is compatible with it (Ellis et al., 2007; Vainio et al., 2011). These mechanisms assist selecting the hand that is the most suitable for the ongoing behavior.

## Supplementary Information

Below is the link to the electronic supplementary material.

Supplementary file1 (XLSX 1205 KB)

Supplementary file2 (DOCX 14 KB)

## Data Availability

Raw anonymized data are included as electronic supplementary material.

## Footnotes

1 Due to COVID-19, data collection was carried out at the homes of participants using a tablet computer.
