# Supplementary material for: Negative affordance effect: automatic response inhibition triggered by handle orientation of non-target object
Source: Psychol Res. 2021 Sep 25;86(6):1737–50. doi: 10.1007/s00426-021-01600-8 (PMC8475350; doi:10.1007/s00426-021-01600-8)
Supplement: Supplementary file 2 — Supplementary file2 (DOCX 14 KB) [file 426_2021_1600_MOESM2_ESM.docx]

README file that defines the coding system and variables in the data set (see Excel file data.xlsx).

1. B3-M31234 (all raw data)

subject: 1-61

freq_block(frequency-block): 1=25%-go, 2=75%-go

comp(compatibility): 1=compatible, 2=incompatible

exp(experiment): 1=one hand, 2=two hands

orientation: 1=left, 2=right

req_resp(required response): 1=left, 2=right, 0=no response

cut_RT: cutted reaction times

RT: uncutted reaction times

response: 1=left, 2=right, 0=no response

correct(data for RT analysis): 1=correct, 2=incorrect

errors(response with wrong hand-data for error analysis): 1=error, 0=no error

false_alarm(data for false alarm analysis): 1=false alarm, 0=no false alarm

1. P3-S122(error%’s for error analysis)
2. V3-Z198(false alarm%’s for false alarm analysis)
